# A Comparison of Retinal Nerve Fiber Layer Thickness and Bruch’s Membrane Opening-Minimum Rim Width Thickness Between Glaucoma and Disc-Suspect Patients Using Optical Coherence Tomography

**DOI:** 10.7759/cureus.92905

**Published:** 2025-09-22

**Authors:** Akash Sidana, Saswati Sen, Matuli Das

**Affiliations:** 1 Ophthalmology, Kalinga Institute of Medical Sciences, Bhubaneswar, IND

**Keywords:** bruch’s membrane, diagnosis, glaucoma, minimum rim width, optical coherence tomogram, retinal nerve fiber layer

## Abstract

Background

Glaucoma results in optic neuropathy, which is irreversible but can be treated effectively when diagnosed early. Several diagnostic parameters have been used to detect the functional and structural damage in glaucoma, which may go hand in hand or precede each other. Therefore, studying the functional and structural changes together may effectively improve the sensitivity and specificity of the diagnosis. In this study, we aim to compare Bruch’s membrane opening-minimum rim width (BMO-MRW) and retinal nerve fiber layer thickness (RNFLT) and assess their role in the diagnosis and prognosis of glaucoma.

Purpose

To compare RNFLT and BMO-MRW values between glaucoma and disc-suspect patients.

Methods

This is a comparative cross-sectional study involving 87 glaucoma patients and 88 disc suspects. A comprehensive ocular examination was performed for all patients, including best-corrected visual acuity (BCVA), intraocular pressure (IOP), and cup-to-disc ratio (CDR) assessment. Optical coherence tomography (OCT) was performed in all cases. We measured RNFLT and BMO-MRW in all quadrants. We used the Mann-Whitney test for intergroup and intragroup comparison, with a p-value <0.05 considered significant.

Results

The mean age was 57 years in the glaucoma group and 42 years in the disc suspect group. The differences in BCVA and CDR between the two groups were statistically significant. Most patients in both groups had BCVA between 6/12 and 6/6, and CDR between 0.5 and 0.7. The mean IOP was 19 mmHg in the glaucoma group, while it was 16 mmHg in disc suspects. Intragroup median RNFLT (glaucoma: 76 µm, disc suspects: 101 µm) and BMO-MRW (glaucoma: 202 µm, disc suspects: 254 µm) showed a significant difference in both groups. Intergroup comparison also showed a positive agreement between both parameters. RNFLT showed better predictability of associated visual field loss than BMO-MRW. In contrast, BMO-MRW was better at detecting subtle structural changes in the retina with more precision than RNFL.

Conclusion

BMO-MRW measurements are positively associated with RNFLT. This helps in diagnosing glaucoma at an early stage and detecting its progression early as well. The technique of measuring BMO-MRW is noninvasive, which gives a more precise measurement of the neuroretinal rim. A combination of both parameters can help in early diagnosis as well as predict the early progression of glaucoma in patients susceptible to developing it.

## Introduction

Glaucoma is a progressive optic neuropathy characterized by loss of retinal ganglion cells, thinning of the retinal nerve fiber layer, and progressive optic disc [1,2]. Functional changes accompany this structural damage, starting with loss of the peripheral fields that progresses to affect the central fields [3]. Both structural and functional changes can occur either concurrently or consecutively. Due to its silent nature, glaucoma is often diagnosed at a stage when damage has already started and is irreversible [4]. Therefore, diagnosing the disease early leads to better prevention and better prognosis [5].

Several strategies and investigations can facilitate the diagnosis of glaucoma cases. In the present scenario, the use of the latest instruments to detect early structural changes and combining it with functional changes helps detect and minimize the damage at an early stage [6]. Optical coherence tomography (OCT) is an effective tool that helps measure optic nerve head and retinal parameters with good precision. It is known that retinal ganglion cell death occurs even before it is apparent to the patient in terms of visual damage [7]. Therefore, the extent of damage can be judged by measuring the loss of retinal nerve fiber layer thickness (RNFLT).

Measuring the loss of the ganglion cell complex (GCC) has also been found to be a major indicator of the extent of damage in glaucoma [8]. In several recent studies, researchers have now found the Bruch’s membrane opening-minimum rim width (BMO-MRW) measurement to be an indicator of such damage as well [9-10]. Its lower susceptibility to external variables provides a more accurate assessment of the structural damage. Therefore, monitoring this parameter not only helps diagnose glaucoma but also helps differentiate glaucomatous optic neuropathy from other causes of disc pallor [11].

In this study, we aim to measure these parameters in both disc suspects and glaucoma patients and determine the diagnostic and prognostic value of these investigations in a population of eastern Odisha in India.

## Materials and methods

The study was a comparative cross-sectional study. We obtained the Institutional Ethical Committee approval (KIIT/KIMS/IEC/1158) and planned the study in a tertiary care setting at the Kalinga Institute of Medical Sciences, Bhubaneswar. The study adhered to the ethical principles of the Declaration of Helsinki. The study period was from December 2023 to November 2024.

Purposive sampling was used, and we included 175 patients for analysis in the study. The glaucoma group had 87 patients and the disc suspect group 88 patients. All these patients reported to the ophthalmology outpatient department of a tertiary care center in the Kalinga Institute of Medical Sciences. We explained the study to eligible patients and received informed consent in writing.

The patient’s demographic profile was recorded. Their proper clinical history was taken, and all patients were subject to slit lamp examination. Parameters such as visual acuity and intraocular pressure (IOP) were measured using applanation tonometry, gonioscopy, fundoscopy, and perimetry, and an OCT examination was conducted to measure RNFLT and BMO-MRW.

The study included glaucoma and disc suspect patients above 18 years of age. Disc suspect patients were defined as those with a cup-disc ratio (CDR) >0.5 in at least one eye or a difference of 0.2 in CDR between the two groups and IOP less than 20 mmHg. We included newly diagnosed or established glaucoma patients under medication in the glaucoma group without any corneal edema to eliminate any bias related to field testing or OCT evaluation. We excluded patients with associated hypertension, dyslipidemia, diabetes mellitus, pre-existing RNFLT or GCC thinning due to ocular diseases other than glaucoma, any corneal or other pathologies hindering the visualization of the fundus and secondary glaucoma.

Snellen’s chart and LogMAR chart were used to record visual acuity. Slit lamp biomicroscopy was employed for the examination of the anterior segment structures and dilated fundus examination was done with +90 D lens. Indirect ophthalmoscopy of the posterior segment was done with +20 D lens to rule out retinopathies, retinal detachment or any other causes of visual defects. Goldmann applanation tonometer was used to record the IOP and perimetry was carried out using Humphrey’s automated perimeter (Carl Zeiss, Oberkochen, Germany). OCT was performed with a spectral domain OCT machine using a BMO-MRW program (Heidelberg Spectralis OCT system equipped with the Glaucoma Module Premium Edition (GMPE), Heidelberg Engineering GmbH, Heidelberg, Germany).

Statistical analysis

Continuous data are presented as mean±standard deviation, and categorical data as frequency with percentage. Wherever applicable, we used the independent t-test and analysis of variance (ANOVA) to compare the average between the two and three groups, respectively. We used IBM SPSS Statistics version 25.0 software (IBM Corp., Armonk, NY, USA) for data collection, tabulation and statistical analysis. A p-value <0.05 was considered to be statistically significant.

## Results

In this cross-sectional study, 175 patients were enrolled, out of which 87 patients were grouped under the glaucoma group and 88 under the disc suspect group. The right eye measurements were considered in both groups to avoid bias. The mean age was 57 years in the glaucoma group, and 42 years in the disc suspect group. The number of men (53; 60.9%) was greater in the glaucoma group as compared to the disc suspect group (46; 52.3%), whereas there were more women in the disc suspect group than the glaucoma group. However, we found this difference to be statistically insignificant. IOP was within normal range for both groups. The best-corrected visual acuity (BCVA) was better in the disc suspects (0.1±0.2) than in glaucoma patients (0.4±0.3). The patients with CDR were more in the range of 0.5-0.7 in both groups, but there were more glaucoma patients (31; 35.6%) with CDR>0.7 as compared to disc suspects (5, 5.8%). This difference was statistically significant in our study. Fields in the Humphrey’s automated perimeter were tested and patients were caterogized as mild (<6 dB), moderate (6-12 dB) and severe (> 12 dB) as per the visual field index in the analysis. The mean BMO-MRW and RNFLT values were greater in the disc suspect group, which was statistically significant. The details are presented in Table 1.

**Table 1:** Baseline values of basic parameters ˟Independent t-test, ^#^Chi-square test, and ^$^Fisher’s exact test. IOP: Intraocular pressure, BCVA: best-corrected visual acuity, RNFL: retinal nerve fiber layer, BMO-MRW: Bruch’s membrane opening-minimum rim width, SD: standard deviation

Parameters		Glaucoma(N=87)	Disc Suspects(N=88)	Test statistic	p-value
Mean age (in years) (mean±SD)		57 ±16	42 ± 12	6.977	<0.001˟
Gender, N (%)	Male	53 (60.9%)	46 (52.3%)	1.331	0.249^#^
	Female	34 (39.1%)	42 (47.7%)		
IOP (in mmHg) (mean±SD)		19 ± 5	16 ± 4	5.688	<0.001˟
BCVA (in LogMar) (mean±SD)		0.4 ± 0.3	0.1 ± 0.2	7.434	<0.001˟
Cup-disc ratio, N (%)	<0.5	0	1 (1.1%)	26.013	<0.001^$^
	0.5-0.7 years	56 (64.4%)	82 (94.2%)		
	>0.7 years	31 (35.6%)	5 (5.8%)		
Field loss, N (%)	Mild	46 (52.8%)	88 (100%)	64.486	<0.001^$^
	Moderate	34 (39.1%)	0		
	Severe	7 (8.1%)	0		
Mean RNFL(in µm) (mean±SD)		76±19	101± 18	-9.011	<0.001˟
Mean BMO-MRW (in µm) (mean±SD)		202±49	254±52	-6.886	<0.001˟

Six quadrants - nasal, temporal, superonasal, superotemporal, inferonasal and inferotemporal - were mapped in the OCT printout and analyzed individually between both groups for the RNFLT and BMO-MRW parameters. The mean RNFLT was found to be significantly lower in the glaucoma group (76±19 µm) than the disc suspect group (101±18 µm). The mean BMO-MRW was also lower in the glaucoma group (202±49 µm) than the disc suspect group (254±52 µm). We found the RNFLT to be highest in the inferotemporal quadrant for both groups. The thickness of all quadrants was higher in the disc suspect group, and the difference was statistically significant when compared with the glaucoma group. While measuring BMO-MRW, we found the thickness was maximum in the inferonasal group of the glaucoma patients, while it was almost similar in the superonasal and inferonasal quadrants in the disc suspect group. The difference in thickness of all quadrants was statistically significant between both the groups. The details are presented in Table 2.

**Table 2:** Quadrantwise comparison between RNFL and BMO-MRW Independent t-test used to calculate the p-value. RNFLT: Retinal nerve fiber layer thickness, BMO-MRW: Bruch’s membrane opening-minimum rim width, SD: standard deviation.

Parameters		Glaucoma (n=87) (mean±SD)	Disc Suspects (n=88) (mean±SD)	t	p-value
RNFL (in µm)	Nasal	67±18	87±18	-7.349	<0.001
	Temporal	55±13	69±13	-6.874	<0.001
	Superonasal	88±29	122±33	-7.323	<0.001
	Superotemporal	102±36	133±26	-6.922	<0.001
	Inferonasal	76±19	112±26	-6.433	<0.001
	Inferotemporal	102±36	140±30	-7.597	<0.001
BMO-MRW (in µm)	Nasal	225±58	284±67	-6.239	<0.001
	Temporal	149±40	181±47	-4.807	<0.001
	Superonasal	216±59	286±72	-7.048	<0.001
	Superotemporal	184±52	248±56	-7.829	<0.001
	Inferonasal	239±65	289±63	-5.200	<0.001
	Inferotemporal	205±76	266±54	-6.068	<0.001

Tables 3, 4 describe the co-relation between IOP, RNFLT and BMO-MRW between the glaucoma and disc suspect groups, respectively. In the glaucoma group, IOP is negatively correlated with both BMO-MRW and RNFLT, which means the thickness of BMO-MRW and RNFL decreases with an increase in IOP. BMO-MRW and RNFLT have a positive correlation, but it is not statistically significant for glaucoma patients. Similarly, in the disc suspects, the IOP has a negative correlation with BMO-MRW and RNFLT, while both BMO-MRW and RNFLT correlate positively. However, we found that the correlation between BMO-MRW and RNFLT is statistically significant in the disc suspects, which may hold some diagnostic and prognostic value.

**Table 3:** Correlation of IOP, RNFL and BMO-MRW within glaucoma cases IOP: Intraocular pressure, RNFL: retinal nerve fiber layer, BMO-MRW: Bruch’s membrane opening-minimum rim width **Correlation is significant at the 0.01 level (two-tailed).

Parameters		IOP (in mmHg)	RNFL (in µm)	BMO-MRW (in µm)
IOP (in mmHg)	Pearson correlation	1	-0.109	-0.182
	p-Value (two-tailed)		0.315	0.091
	N	87	87	87
RNFL (in µm)	Pearson correlation	-0.109	1	0.691^**^
	p-Value (two-tailed)	0.315		0.000
	N	87	87	87
BMO-MRW (in µm)	Pearson correlation	-0.182	0.691^**^	1
	p-Value (two-tailed)	0.091	0.000	
	N	87	87	87

**Table 4:** Correlation of IOP, RNFL and BMO-MRW within disc suspects IOP: Intraocular pressure, RNFL: retinal nerve fiber layer, BMO-MRW: Bruch’s membrane opening-minimum rim width **Correlation is significant at the 0.01 level (two-tailed).

Parameters		IOP (in mmHg)	RNFL (in µm)	BMO-MRW (in µm)
IOP (in mmHg)	Pearson correlation	1	-0.146	-0.228^*^
	p-Value (two-tailed)		0.175	0.033
	N	88	88	88
RNFL (in µm)	Pearson correlation	-0.146	1	0.606^**^
	p-Value (two-tailed)	0.175		0.000
	N	88	88	88
BMO-MRW (in µm)	Pearson correlation	-0.228^*^	.606^**^	1
	p-Value (two-tailed)	0.033	0.000	
	N	88	88	88

Table 5 summarizes the descriptives within the glaucoma group for finding the association of Humphrey field analysis (HFA) with RNFLT and BMO-MRW. Most of the patients (46; 52.8%) had mild field loss, followed by moderate (34; 39.1%) and severe (7; 8.1%) field loss. This was statistically significant in association with RNFLT and BMO-MRW both for changes within the groups or between the groups. Thus, we can conclude that the changes in functional and structural parameters go hand in hand for patients who have glaucoma. Because the disc suspects had no or mild visual field changes in our study, we noted no significant change in the pattern of field loss in association with the structural changes (RNFLT and BMO-MRW). The magnitude of change in RNFLT, therefore, was steeper compared to BMO-MRW when compared to the visual field changes.

**Table 5:** Association of visual field loss with RNFL and BMO-MRW within glaucoma cases Analysis of variance (ANOVA) test used for calculation. RNFL: Retinal nerve fiber layer, BMO-MRW: Bruch’s membrane opening-minimum, SD: standard deviation.

Parameters	Visual field loss	N (%)	Mean±SD	95% Confidence interval for mean		F	p-value
				Lower Bound	Upper Bound		
RNFL (in µm)	Mild	46(52.8%)	82.40±15.753	77.67	87.13	33.873	<0.001
	Moderate	34(39.1%)	74.00±17.564	67.87	80.13		
	Severe	7(8.1%)	47.43±17.203	31.52	63.34		
	Total	87(100%)	76.23±18.962	72.17	80.30		
BMO-MRW) (in µm)	Mild	46(52.8%)	224.13±40.650	211.92	236.35	31.623	<0.001
	Moderate	34(39.1%)	187.65±42.609	172.78	202.51		
	Severe	7(8.1%)	135.71±35.349	103.02	168.41		
	Total	87(100%)	202.51±48.495	192.11	212.91		

## Discussion

RNFLT and BMO-MRW are often correlated, though factors such as blood vessel location and the specific anatomy of the optic disc may cause some differences. Therefore, they may not at all times indicate a similar degree of optic nerve head damage. The thickness of the nerve fiber layer itself gives the measurement of RNFL, while measuring the distance from the opening of the Bruch’s membrane to the internal limiting membrane gives the value of the BMO-MRW. Put simply, BMO-MRW measures the smallest distance between these two given endpoints [12]. In our study, we took circle scans into consideration for both measurements [13]. As expected, the baseline parameters were better in the disc suspects. Because most patients had mild field loss and cupping between 0.5 and 0.7, the results provide some insight into the early pattern of changes in the development of glaucomatous damage.

We compared the measurements of the six sectoral quadrants in RNFLT and BMO-MRW both within the two groups and between the two groups. While RNFL was thicker in the inferotemporal quadrant, BMO-MRW measurements were thicker in the superonasal and inferonasal quadrants. This implied that in certain cases, when both clinically and functionally correlated, the subtle glaucomatous damage in various quadrants can be picked up early if both RNFLT and BMO-MRW are considered for diagnosis. At the same time, discrepancies such as these can also help eliminate the false positives. Several researchers have explored the diagnostic abilities of RNFLT and BMO-MRW and found that, in spite of some sectoral disagreements between both parameters, combining both may aid in the early diagnosis of glaucoma [14,15]. Some researchers have also reported that BMO-MRW is more effective in ruling out glaucoma in cases of myopic discs or macro discs [16,17].

In concurrence with the results of studies done by Gietzelt et al. [18] and Pardon et al. [19], we also observed a negative correlation of IOP with BMO-MRW and RNFLT. This implies that with better control of IOP, the thinning of BMO-MRW and RNFLT can be prevented, although the same may not be seen in the transient change in IOP. We also found that RNFLT and BMO-MRW were positively correlated, with the rate of change being statistically significant between the glaucoma and disc suspect groups. This implies that these parameters can help predict early detection and progression of glaucoma.

Structural and functional changes in glaucoma may not go hand in hand [20,21]. Therefore, detecting the structural damage prior to detectable field loss is paramount in controlling glaucoma [22,23]. Researchers compared the structural and visual changes in glaucoma and noted that, for the patient to detect visual field loss, a sizable degree of BMO-MRW loss must be present [24]. Similarly, in our study, most of the disc suspects had no or mild visual field loss. Thus, we found no significant association between visual field loss and BMO-MRW. However, in the glaucoma group, the rate of change of visual field loss ranging from mild to moderate and then to severe was significant in association with both RNFLT and BMO-MRW. La Bruna et al. found a strong association of changes in BMO-MRW thickness with corresponding visual fields [25]. Our finding implies that both parameters can aid in detecting the progression of glaucoma. The degree of change in RNFLT is steeper than BMO-MRW when compared against the visual field. This implies that the same field changes will cause a bigger change in the RNFLT value than that of BMO-MRW. Therefore, according to our study, RNFLT has better predictability in detecting field changes than BMO-MRW. However, BMO-MRW has been proved to be more effective than RNFLT measurement when studying people with myopic or abnormal discs and those with disc hemorrhages with an imminent risk of glaucoma [26-28].

The BMO-MRW values in the Eastern Indian population of the present study are similar to studies that compared the measurement of BMO-MRW between different races in Brazil [29]. Our study emphasizes the importance of both RNFLT and BMO-MRW parameters in both the detection and progression of glaucoma.

Limitations

Further longitudinal studies measuring the changes in values of RNFLT and BMO-MRW over time will give a better idea of the diagnostic value of these tests. In addition, segmentation errors may have led to the omission of some sectoral errors while averaging the values of RNFLT and BMO-MRW.

## Conclusions

RNFLT and BMO-MRW both highlight different perspectives on the neuroretinal rim. We can conclude that both RNFLT and BMO-MRW are of paramount importance in glaucoma-related optic neuropathies. Assessing both values together will increase the specificity of the diagnosis, whereas considering either one, while comparing it with the other, will increase the sensitivity. In addition, combining these findings with the visual field changes will improve the prognostic and diagnostic ability of the tests. Therefore, incorporating BMO-MRW measurement into our daily practice will not only help different other optic neuropathies from glaucoma but will help prevent visual loss due to glaucoma. To our knowledge, this is the first study to evaluate and compare BMO-MRW values alongside RNFLT in glaucoma and disc suspects in a subset of the Indian population. Longitudinal follow-up studies will help establish the relation between these parameters better and help in exploring the diagnostic and prognostic advantage of these parameters in the Indian population.
